# The TOR Signaling Network in the Model Unicellular Green Alga *Chlamydomonas reinhardtii*

**DOI:** 10.3390/biom7030054

**Published:** 2017-07-12

**Authors:** María Esther Pérez-Pérez, Inmaculada Couso, José L. Crespo

**Affiliations:** Instituto de Bioquímica Vegetal y Fotosíntesis, Consejo Superior de Investigaciones Científicas (CSIC)-Universidad de Sevilla, Avda. Américo Vespucio 49, Sevilla 41092, Spain; eperez@ibvf.csic.es (M.E.P.-P.); inmaculada.couso@ibvf.csic.es (I.C.)

**Keywords:** target of rapamycin (TOR), rapamycin, FKBP12, *Chlamydomonas*, algae, autophagy, lipid metabolism

## Abstract

Cell growth is tightly coupled to nutrient availability. The target of rapamycin (TOR) kinase transmits nutritional and environmental cues to the cellular growth machinery. TOR functions in two distinct multiprotein complexes, termed TOR complex 1 (TORC1) and TOR complex 2 (TORC2). While the structure and functions of TORC1 are highly conserved in all eukaryotes, including algae and plants, TORC2 core proteins seem to be missing in photosynthetic organisms. TORC1 controls cell growth by promoting anabolic processes, including protein synthesis and ribosome biogenesis, and inhibiting catabolic processes such as autophagy. Recent studies identified rapamycin-sensitive TORC1 signaling regulating cell growth, autophagy, lipid metabolism, and central metabolic pathways in the model unicellular green alga *Chlamydomonas reinhardtii*. The central role that microalgae play in global biomass production, together with the high biotechnological potential of these organisms in biofuel production, has drawn attention to the study of proteins that regulate cell growth such as the TOR kinase. In this review we discuss the recent progress on TOR signaling in algae.

## 1. Introduction

The amount and quality of nutrients regulate cell growth in all living organisms. In eukaryotes, the availability of nutrients is sensed through different signaling networks such as the TOR (target of rapamycin) kinase. Since its identification in the budding yeast *Saccharomyces cerevisiae* [1], the study of the TOR signaling network expanded to other systems, including mammals, insects, protozoa, and plants [2,3,4,5]. The TOR kinase plays an essential and conserved role in integrating nutritional and energy inputs for the proper regulation of cell growth in lower and higher eukaryotes. TOR controls cell growth by promoting a number of anabolic processes, including translation, ribosome biogenesis, and transcription, and by antagonizing catabolic processes such as autophagy and mRNA degradation [3,6]. Biochemical and genetic studies in yeast demonstrated that TOR regulates these important cellular processes by two independent signaling branches defined by the TOR complex 1 (TORC1) and TOR complex 2 (TORC2) protein complexes [7,8,9,10]. The basic architecture of TORC1 and TORC2 appears to be structurally and functionally conserved in many eukaryotes, although central components of TORC2 have not been identified in plants [2,11]. Evolutionary studies have shown that core components of the TOR signaling network are conserved among divergent species and likely evolved from an archaic pathway of the last eukaryotic common ancestor [12].

The TOR kinase has also been described in algae, indicating an early evolutionary origin of this signaling network in photosynthetic eukaryotes [13]. The study of TOR in these organisms is still in its infancy, although recent findings have contributed to understand how TOR controls cell growth in algae. The first experimental evidence of TOR conservation in these organisms was reported in the model green alga *Chlamydomonas reinhardtii* (referred to here as Chlamydomonas) [13]. More recently, a thorough genomic analysis of several algal species from different clades revealed that the TOR kinase and other TORC1 components are widely conserved in algae [14]. It has been reported that the growth of Chlamydomonas cells is inhibited by rapamycin [13], whereas early studies indicated that some plants are resistant to this drug [15]. However, rapamycin resistance is not a common feature of land plants since it has been reported that the growth of tomato and Arabidopsis seedlings is sensitive to high concentrations of this drug [16,17]. The sensitivity of Chlamydomonas cells to rapamycin, together with the easy manipulation of this organism, its simple life cycle, and a growing array of genetic and molecular tools [18], have positioned this green alga as a convenient model system to investigate the TOR network in photosynthetic eukaryotes.

## 2. Inhibition of TOR Signaling by Rapamycin in Chlamydomonas

**Figure 1:**
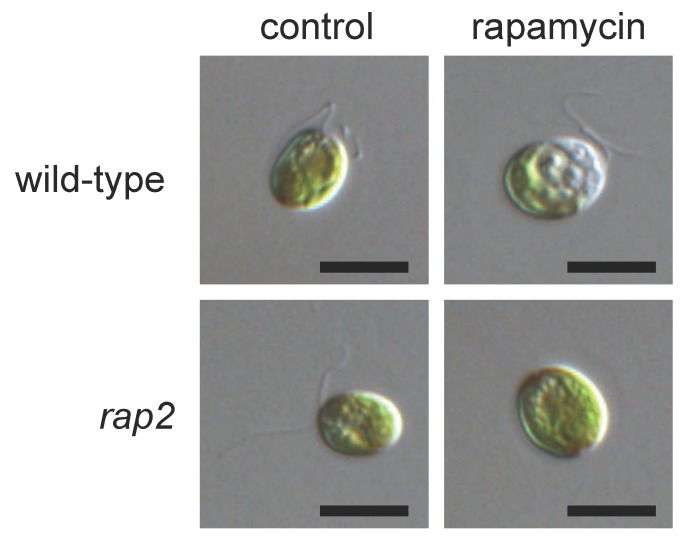
Sensitivity of Chlamydomonas cells to rapamycin. Nomarski images of wild-type and *rap2* mutant cells treated with 500 nM rapamycin for 24 h. Bleaching and pronounced vacuolization is observed in wild-type treated cells but not in the rapamycin-resistant mutant *rap2*, which lacks the FKBP12 (FK506-binding protein of 12 kDa) protein. Scale bar, 10 μm. Adapted from [13].

Rapamycin is an antifungal, secondary metabolite produced by the soil bacterium *Streptomyces hygroscopicus* that was originally identified as a potent immunosuppressive agent [19]. The mechanism of action of rapamycin is unique and both the receptor and functional target were defined genetically in *S. cerevisiae* [1]. Rapamycin first binds the FKBP12 (FK506-binding protein of 12 kDa) immunophilin, and this complex specifically inhibits TOR function by restricting the access of substrates to the kinase domain [20]. Although rapamycin potently inhibits cell growth in most eukaryotes, the vegetative growth of Arabidopsis and other plants such as *Oryza sativa*, *Nicotiana tabacum*, or *Brassica napus* is not sensitive to rapamycin [15] due to the inability of plant FKBP12 to form a stable complex with this drug [17,21,22]. Unlike plants, the growth of the unicellular green alga *Chlamydomonas reinhardtii* is sensitive to rapamycin, indicating the presence of a rapamycin-sensitive TOR signaling cascade in this photosynthetic organism [13]. The treatment of Chlamydomonas cells with rapamycin resulted in increased vacuolization and cell cycle arrest (Figure 1). As described in other eukaryotes, the negative effects of rapamycin in Chlamydomonas cell growth are mediated by FKBP12. The FKBP12 gene is highly conserved in Chlamydomonas, and an evolutionary analysis revealed that Chlamydomonas FKBP12 does not group with plant homologues and is positioned in the same clade as FKBP12 proteins, distinct from yeasts and animals [13]. Sensitivity to rapamycin does not seem to be a common feature conserved among algae since some species such as the red alga *Cyanidioschyzon merolae* are fully resistant to this drug, which is likely due to divergence of FKBP12 protein in this alga [23]. However, the expression of yeast FKBP12 in *C. merolae* cells conferred sensitivity to this drug and allowed the study of rapamycin-sensitive TOR signaling in this alga [24]. Other algae like *Euglena gracilis*, an early branching photosynthetic protist, or *Emiliania huxleyi*, a highly abundant coccolithophore in oceans, might be sensitive to rapamycin (see below), although cell growth does not seem to be inhibited by this drug [25,26].

Chlamydomonas FKBP12 binds rapamycin both in vitro and in vivo, but its affinity to this drug is lower compared to yeast or mammalian FKBP12 proteins [13]. The crystal structure of the human FKBP12-rapamycin complex revealed that rapamycin binds FKBP12 through hydrophobic contacts and hydrogen bonds with key residues in a hydrophobic pocket of the protein [27]. Chlamydomonas FKBP12 has a conserved drug-binding pocket. Except for Gln53 from human FKBP12, all residues that establish hydrogen bonds with rapamycin are conserved in this alga. The absence of this Gln residue in the drug-binding pocket might cause a decreased affinity of Chlamydomonas FKBP12 to rapamycin. This hypothesis was confirmed by the analysis of Chlamydomonas FKBP12 mutants, where Gln53 was artificially generated to mimic the rapamycin-binding pocket of yeast and human FKBP12s [13]. The expression of these mutants in Chlamydomonas resulted in increased sensitivity of the cells to rapamycin [13]. These experiments demonstrated that the low sensitivity of Chlamydomonas cells to rapamycin is mainly due to a decreased affinity of FKBP12 to this drug. A phylogenetic analysis of FKBP12 proteins from different organisms also revealed that some residues that bind rapamycin, including Tyr27 and Asp38 [27], are not conserved in plants [13].

As initially reported in yeasts [1], the identification of a Chlamydomonas mutant lacking the FKBP12 protein provided solid genetic evidence linking rapamycin to TOR signaling in algae [13]. The Chlamydomonas FKBP12-deficient mutant, named *rap2*, exhibited complete resistance to rapamycin and was identified in a screen for spontaneous mutants resistant to this drug. A detailed analysis of this mutant revealed a large DNA reorganization in the *FKBP12* locus that prevented the expression of the *FKBP12* gene [13]. The *rap2* mutant has proven to be a useful tool for the study of the TOR signaling network in Chlamydomonas. Similar mutants might also be identified in other rapamycin-sensitive algae given the high frequency of spontaneous rapamycin-resistant mutants found in Chlamydomonas.

## 3. TOR Complexes in Algae

The TOR kinase is a large protein (about 280 kDa in yeasts, mammals, plants and algae) that assembles into two structurally and functionally distinct multiprotein complexes, termed TOR complex 1 (TORC1) and TOR complex 2 (TORC2). Initially identified in yeasts [9,10], TORC1 and TORC2 were subsequently described in mammals [28,29,30,31,32] and other eukaryotes [33,34,35]. The core components of TORC1 in yeasts and mammals include the TOR kinase, KOG1/Raptor, and LST8, whereas conserved TORC2 is composed of TOR, LST8, AVO1/hSin1, and AVO3/Rictor. In both yeasts and mammals, the FKBP12-rapamycin complex targets and inhibits TORC1 but curiously not TORC2 [9,10,28,29,30,31]. The molecular basis of the rapamycin insensitivity to TORC2 has been recently established in yeasts. A thorough analysis of the molecular architecture of this complex revealed that AVO3, a subunit unique to TORC2, could mask the FKBP12-rapamycin-binding (FRB) domain of TOR2, thereby conferring insensitivity to inhibition by FKBP12-rapamycin [36]. 

The TOR kinase and the TORC1 proteins LST8 and KOG1/Raptor have been identified in Chlamydomonas and are widely conserved in algae from different clades [13,14,37,38]. The Chlamydomonas TOR protein contains highly conserved domains, including the FRB and kinase domains, N-terminal HEAT repeats, and the FAT and FATC domains characteristic of phosphatidylinositol 3-kinase related kinases [13]. A comparative study of TOR proteins from different eukaryotes indicates that the closest homologues to Chlamydomonas TOR are found in plants and green algae [13]. A recent genomic analysis has revealed that TOR is widely conserved in algal genomes, including freshwater and marine species from Chlorophyta, Rhodophyta, and Chromalveolata clades [14]. This study also showed that most algae have a single TOR protein, although some species such as the marine alga *Emiliania huxleyi* encode three TOR kinases [14]. Whether these three TOR proteins are simultaneously expressed in *E. huxleyi* to assemble in a single TOR complex (TORC1) is currently unknown. In this regard, the presence of multiple TOR proteins has been reported in other organisms such as the protozoan parasite *Trypanosoma brucei*, which contains four TOR orthologs, although only two of them display structural features characteristic to TOR proteins [33].

**Figure 2:**
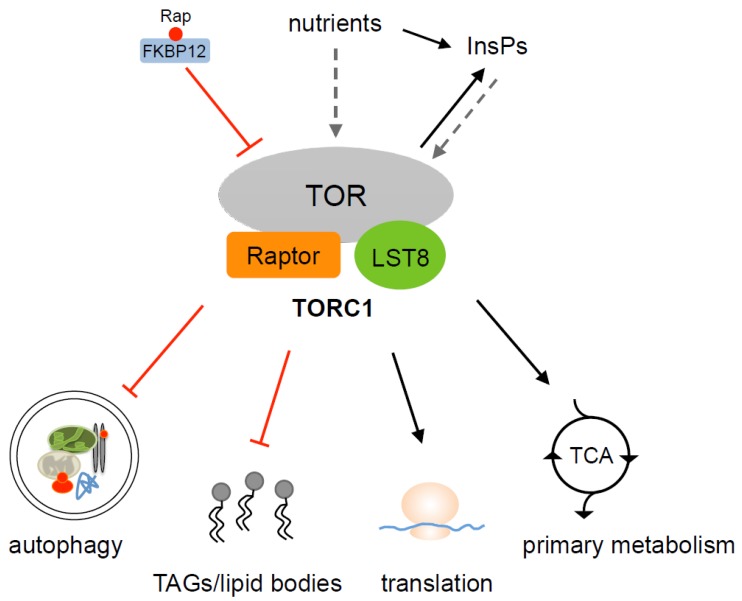
Proposed model of target of rapamycin (TOR) signaling in Chlamydomonas. A rapamycin-sensitive TORC1 complex composed by TOR, LST8, and Raptor/KOG1 is conserved in Chlamydomonas, whereas TORC2 core components are missing in this model alga. Inhibition of Chlamydomonas TORC1 signaling by rapamycin-FKBP12 activates autophagy, promotes the accumulation of TAGs and the formation of lipid bodies, inhibits protein synthesis, and blocks central metabolic pathways such as the TCA cycle. TORC1 may control these processes by perceiving nutrient (carbon, nitrogen) availability and the intracellular level of inositol polyphosphates. Solid lines indicate interactions that have been experimentally demonstrated (red, inhibition), while links represented by dashed arrows need to be tested. Rap, rapamycin; InsPs, inositol polyphosphates; TAGs, triacylglycerols; TCA, tricarboxylic acid.

The presence of TOR in high-molecular-mass complexes has been demonstrated in Chlamydomonas [37]. This alga contains single LST8 and KOG1/Raptor genes, the products of which show high identity to their yeast and mammalian homologs [37]. Chlamydomonas LST8 forms part of a rapamycin-sensitive TORC1 complex since it can be co-purified with TOR and FKBP12 in the presence of rapamycin [37] (Figure 2). Yeast and mammalian LST8s bind to the kinase domain of TOR and this interaction is required for the full catalytic activity of TOR [31,39]. Chlamydomonas LST8 is able to bind to the purified kinase domain of TOR, suggesting that LST8 may perform a similar role in Chlamydomonas. Moreover, yeast complementation assays demonstrated that Chlamydomonas LST8 is able to functionally and structurally replace endogenous yeast LST8, indicating that the function of this protein must be conserved in photosynthetic organisms [37]. The physical interactions of LST8 with TOR in Chlamydomonas may play important roles in regulating the TOR network in this organism since LST8 consists entirely of seven WD-40 domains, which mediate protein-protein interaction. Three residues from Chlamydomonas LST8 that are conserved in yeasts, plants, and algae seem to be required for the function of this protein [37]. Point mutations at positions Asp106, Thr228, or Ser230 of Chlamydomonas LST8 failed to complement an *lst8* mutation in yeasts and prevented the interaction of this protein with TOR. These findings indicated that the interaction of Chlamydomonas LST8 with TOR in yeasts might be required to keep TOR active, which is essential for cell growth.

Biochemical fractionation studies of Chlamydomonas cells revealed that the LST8 and TOR proteins are peripherally associated with internal membranes in this alga, suggesting that TOR complexes may localize on these membranous sites [37]. In both yeasts and mammals, TORC1 has been localized at the surface of the vacuole/lysosome, and this position is needed for its activation (reviewed in [40]). However, yeast and mammalian TORs have also been detected at other sites, including mitochondria, nucleus, plasma membrane, stress granules, or the cytoplasm. This broad distribution of TOR proteins might be due to the different locations of TOR substrates or may just reflect the diversity of methods used to investigate the subcellular localization of TOR complexes [40]. In Chlamydomonas, the TOR and LST8 proteins seem to accumulate at the peri-basal body region and at discrete bodies in the cytoplasm [37]. The peri-basal body region is localized at the flagella base and may represent a region of the cell with a high demand of protein synthesis for the formation of flagella [41]. Why Chlamydomonas TOR and LST8 proteins localize at this site of the cell is currently unknown.

LST8 and KOG1/Raptor have also been identified in the genomes of green, red, and chromalveolate algae, suggesting that TORC1 is widely conserved in these organisms [14]. However, the TORC2-specific proteins AVO1/hSIN1 and AVO3/Rictor seem to be lost in algae and plants [14,38,42], raising the question of whether TORC2 is structurally conserved in photosynthetic organisms. Despite the absence of TORC2 proteins, it is possible that algae and plants may have a TOR complex functionally similar to TORC2 constituted by highly divergent proteins compared to their yeast and mammalian counterparts.

## 4. Control of Autophagy by TOR

Autophagy is a major catabolic pathway by which eukaryotic cells deliver unnecessary or damaged cytoplasmic material to the vacuole for degradation and recycling in order to maintain cellular homeostasis. During this degradative process, cytosolic components, including proteins, membranes, and entire organelles, are engulfed in bulk or selectively within a double-membrane vesicle known as an autophagosome and delivered to the vacuole [43,44]. Autophagy is mediated by autophagy-related (*ATG*) genes, which were originally identified in yeasts and subsequently in other eukaryotes, including mammals, plants, and algae [14,38,45,46,47]. Autophagy is triggered among other factors by a reduction in the availability of nutrients. TORC1 was identified in yeasts as a malor regulator of this catabolic pathway that signals nutrient availability to the autophagic machinery [48]. Under nutrient-rich conditions, TORC1 phosphorylates ATG13, a regulatory subunit of ATG1, and this phosphorylation prevents the activation of ATG1 and hence autophagy [49]. However, nutrient limitation or the inhibition of TORC1 by rapamycin causes rapid dephosphorylation of ATG13, leading to ATG1 activation and autophagy induction. The molecular mechanism of autophagy regulation by TORC1 seems to be conserved in metazoans through the direct phosphorylation of the ATG1 and ATG13 proteins [50].

The initial characterization of Chlamydomonas cells treated with rapamycin led to the hypothesis that TOR controls autophagy in photosynthetic eukaryotes based on the observation that rapamycin-treated cells exhibited increased vacuolization and bleaching [13] (Figure 1). This conclusion was confirmed with the generation of specific autophagy markers in Chlamydomonas. The autophagy machinery is well conserved in this model alga, and ATG genes are present as single copy genes in the Chlamydomonas genome [38]. An evolutionary analysis of autophagy genes in different algae revealed that core ATG genes are highly conserved in the green plastid lineage but, surprisingly, not in red algae [14,46]. The establishment of the ATG8 protein as a specific autophagy marker in Chlamydomonas has demonstrated that indeed this catabolic process occurs in green algae, and it is inhibited by a rapamycin-sensitive TOR signaling network [51,52] (Figure 2). ATG8 is a highly conserved protein that binds to the autophagosome membrane and remains linked to the mature autophagosome until this specialized vesicle fuses with the vacuole [53]. The association of ATG8 to the autophagosome occurs through the covalent binding of the lipid phosphatidylethanolamine to a conserved C-terminal Gly residue of this protein in a process known as ATG8 conjugation or lipidation. The activation of autophagy is usually monitored through the detection of lipidated ATG8 forms and changes in the cellular localization of this protein [54]. In Chlamydomonas, rapamycin treatment led to detection of lipidated ATG8 as well as an increase in the abundance of this protein [52]. The cellular localization of ATG8 was also altered in response to rapamycin treatment. Under favorable growth conditions, the ATG8 signal is weak and limited to various punctate structures. However, the induction of autophagy by rapamycin treatment drastically modified the cellular localization of Chlamydomonas ATG8, and larger spots were detected throughout the cytoplasm. These results demonstrated that indeed a rapamycin-sensitive TORC1 pathway controls autophagy in Chlamydomonas [52]. It remains to be determined whether, as reported in other systems [50], the inhibition of TOR by rapamycin triggers autophagy through the activation of the ATG1 kinase, which is conserved in Chlamydomonas [38]. Rapamycin has also been used as an autophagy inducer in the coccolithophore *Emiliania huxleyi*, indicating that the control of autophagy by TOR might be conserved in evolutionarily distant algae [26]. Moreover, the inhibition of autophagy by TOR seems to be conserved in the green lineage since it has been shown that Arabidopsis mutant lines with decreased TOR expression display constitutive autophagy [55].

The study of autophagy in Chlamydomonas has revealed that this degradative process is induced by nutrient starvation [51,52]. Nitrogen or carbon limitation in Chlamydomonas resulted in a significant increase in ATG8 protein abundance as well as the detection of modified forms of ATG8, both landmarks of autophagy activation. Moreover, as described in rapamycin-treated cells, ATG8 was detected in punctate structures in nitrogen-limited Chlamydomonas cells [52]. In addition to nutrient starvation, autophagy is induced in Chlamydomonas in response to oxidative stress or the accumulation of unfolded proteins in the endoplasmic reticulum (ER stress) [51,52,56]. These stress conditions resulted in increased ATG8 abundance, the detection of modified ATG8, and a drastic change in the cellular localization of this protein. Given the central role of the TOR kinase in perceiving and transmitting nutrient and stress signals to the growth machinery in eukaryotes, TOR might likely participate in the control of autophagy in response to nutrient deficiency or ER stress in Chlamydomonas, although this has not yet been demonstrated.

## 5. TOR Promotes Protein Synthesis in Chlamydomonas

The control of protein synthesis is one of the best-characterized processes downstream of TOR. In yeasts and mammals, TORC1 promotes protein synthesis by activating translation and ribosome biogenesis (reviewed in [57,58]). TORC1 directly phosphorylates the key regulators of protein synthesis such as mammalian S6K or yeast SCH9 kinases. Under optimal growth, yeast TORC1 phosphorylates the C terminus of SCH9, and phosphorylated SCH9 participates in the activation of all three RNA polymerases [59]. In mammalian cells, phosphorylated S6K promotes the phosphorylation of the ribosomal protein S6, which in turn up-regulates translation initiation [60]. This branch of the TORC1 network is conserved in plants. The Arabidopsis genome encodes two S6K homologs, S6K1 and S6K2 [61], and it has been demonstrated that TOR regulates S6K1 and S6 phosphorylation in Arabidopsis [17,62,63]. In Chlamydomonas, it has been shown that rapamycin inhibits protein synthesis [64], although it is currently unknown whether a TOR-S6K pathway may operate in this model alga to regulate protein synthesis. Nevertheless, the ER chaperone BiP was identified in a screen for TOR targets in Chlamydomonas as a protein with a phosphorylation state that is indirectly regulated by TOR through the control of protein synthesis [64]. The treatment of Chlamydomonas cells with rapamycin or cycloheximide resulted in the inhibition of protein synthesis and the phosphorylation of BiP. Interestingly, BiP phosphorylation was prevented in Chlamydomonas under conditions that require activation of BiP, such as tunicamycin treatment or heat shock stress, which inhibits *N*-linked glycosylation of nascent proteins in the ER [64]. These results also highlighted the important role that the control of protein synthesis by TORC1 plays in the regulation of ER homeostasis, as previously reported in mammals [65].

## 6. TOR Signaling and Lipid Metabolism in Algae

The discovery that TOR inactivation by rapamycin elicits a nutrient starvation response demonstrated that TOR is part of a signaling network coupling nutrient cues to cell growth [66]. In yeasts, TOR signaling responds to nitrogen availability, and, accordingly, nitrogen limitation and rapamycin trigger a similar starvation response [67,68,69]. An interesting observation has been recently reported linking TOR signaling and nitrogen starvation in algae. In response to nitrogen limitation, these organisms synthesize large amounts of triacylglycerols (TAGs) that are accumulated in specialized structures known as lipid bodies [70,71,72,73]. The inhibition of TOR signaling by rapamycin, AZD8055, or Torin1 in Chlamydomonas and the distant red alga *Cyanidioschyzon merolae* resulted in the accumulation of lipid bodies containing TAGs and the up-regulation of genes involved in TAG synthesis such as glycerol-3-phosphate acyltransferase and acyl-CoA:diacylglycerol acyltransferase (DGAT) [24,74]. A similar effect on lipid body accumulation has also been shown in the green alga *E. gracilis* treated with rapamycin [25]. Together, these observations indicate that TOR may play an important role in the control of lipid metabolism and TAG synthesis (Figure 2), in agreement with previous studies in yeasts showing that TORC1 and TORC2 maintain the homeostasis of lipid metabolism by regulating the synthesis of triacylglycerol, sphingolipids, and very long chain fatty acids [75,76,77].

The molecular mechanisms underlying the regulation of lipid storage by TOR in algae are currently unknown, although significant progress in this field has been recently shown in Chlamydomonas. In a screen for rapamycin-hypersensitive mutants in this model alga, a loss-of-function mutation in VIP1, a gene encoding a conserved inositol polyphosphate kinase that pyrophosphorylates InsP_6_ to produce the signaling molecules InsP_7_ and InsP_8_, was identified [78]. Levels of InsP_7_ and InsP_8_ were decreased in *vip1-1* mutant cells, suggesting that InsP_7_ and InsP_8_ are required for cell growth in conjunction with TOR kinase activity. Whether inositol polyphosphates (InsPs) may act upstream, downstream, or in a parallel pathway to TOR to control cell growth in Chlamydomonas is unknown, but the observation that the treatment of wild-type cells with rapamycin leads to changes in InsPs levels suggests that these molecules might be downstream of TOR signaling [78] (Figure 2). Interestingly, *vip1-1* mutant cells exhibit increased levels of TAGs and a higher amount of lipid bodies, even under conditions in which lipid bodies are usually low in wild-type cells [78], indicating an interaction between InsPs, lipid metabolism, and TOR. Growing attention has been focused on the study of the metabolic pathways of Chlamydomonas, leading to the accumulation of TAGs due to the biotechnological potential of these lipids for biofuel production [79,80]. Given the role of TOR in the control of lipid metabolism and TAGs storage in microalgae, this signaling pathway might also be a biotechnological target to improve TAG productivity in algae.

## 7. Control of Primary Metabolism by TOR in Chlamydomonas

The sensitivity of Chlamydomonas to rapamycin has been considered an experimental advantage to explore the function of TOR in a photosynthetic organism, and transcriptomic and metabolomic analyses of Chlamydomonas cells treated with this drug have been recently reported. Metabolomic studies revealed that the tricarboxylic acid (TCA) cycle is largely affected by the inhibition of TOR (Figure 2), and some TCA cycle intermediates including malate, succinate, and citrate accumulated in rapamycin-treated cells [81,82]. The negative effect on the TCA cycle is accompanied by the simultaneous down-regulation of some amino acids such as phenylalanine, glutamic acid, aspartic acid, asparagine, tyrosine, or glutamine [82]. It has been proposed that the decrease of these amino acids in rapamycin-treated cells can be associated with the activation of nutrient recycling processes such as senescence or autophagy [82], which are activated by rapamycin in Chlamydomonas [52]. Thus, TOR seems to play a prominent role in the control of primary metabolism in Chlamydomonas, as previously reported in Arabidopsis mutant plants with decreased TOR expression [83]. Remarkably, metabolomics data also indicated that cysteine and methionine pools are strongly affected in Chlamydomonas cells treated with rapamycin [81]. A cysteine and methionine metabolism is needed for the assimilation of sulfur and the synthesis of glutathione, a highly abundant free soluble thiol that maintains the intracellular redox balance in the cell [84]. The altered metabolism of cysteine and methionine in rapamycin-treated cells suggests that proper TOR function might be required to maintain redox homeostasis. In close agreement with this hypothesis, it has been shown that the down-regulation of TOR in Arabidopsis results in the enhanced synthesis of glutathione [83,85]. Finally, a metabolomic analysis of Chlamydomonas cells treated with rapamycin also showed a depletion of key intermediates in glycolysis, the pentose phosphate pathway, and nucleotides, as well as an activation of the proline pathway, which is a typical metabolic phenotype under nitrogen limitation [82].

Transcriptomic studies performed in Chlamydomonas cells treated with rapamycin demonstrated that the inhibition of TOR signaling had a profound effect on the expression of several thousand genes [81,86]. Up-regulated genes are implicated in amino acid metabolism, vacuolar function, tetrapyrrole metabolism, autophagy, and the transport of metabolites. Most highly induced genes include small heat shock proteins and chaperones, proteases, proteins involved in autophagy and thylakoid membrane biogenesis, protein kinases, and transporters [86]. Genes with transcripts that are down regulated upon rapamycin treatment are involved in cell cycle, DNA replication and repair, nucleotide metabolism, and photosynthesis [86]. Together, these transcriptomic and metabolomic data highlight the central role of TOR in cell growth control by regulating anabolic and catabolic processes in Chlamydomonas, as previously described in other eukaryotes [2,3,4,11].

## 8. Perspectives

Our current knowledge about TOR signaling in algae is limited compared to other systems. Despite recent advances in this field, little is known about the molecular mechanisms by which TOR promotes photosynthetic cell growth. Efforts should be made on the identification of the upstream and downstream components of this signaling cascade and the establishment of a TOR kinase assay, which is not currently available for Chlamydomonas. Although a rapamycin-sensitive TORC1 pathway has been shown to regulate autophagy in this model alga [52], it remains to be investigated whether other TORC1 readouts such as the control of ribosome biogenesis or the transcriptional regulation of central metabolic pathways are conserved in algae. Carbon assimilation is critical for cell growth in photosynthetic organisms, and, given the central role of TOR in nutrient signaling, a model in which this kinase integrates signals from carbon metabolism to promote cell growth is feasible [87]. The identification of TOR as an important regulator of lipid storage and InsPs metabolism in algae [24,78] opens new and promising research lines in the TOR field that might be extended to higher plants. Moreover, a better knowledge of how TOR integrates nutritional cues and regulates central metabolism in algae may redound to benefits in industrial sectors like biofuel production due to the high potential of these organisms as biofuel precursors [79,80].
